# Monophasic transcranial constant-current versus constant-voltage stimulation of motor-evoked potentials during spinal surgery

**DOI:** 10.1038/s41598-019-39883-y

**Published:** 2019-03-07

**Authors:** Keisuke Masuda, Hideki Shigematsu, Masato Tanaka, Eiichiro Iwata, Yusuke Yamamoto, Masahiko Kawaguchi, Tsunenori Takatani, Sachiko Kawasaki, Yasuhito Tanaka

**Affiliations:** 1 Department of Orthopaedic Surgery, Nara Medical University, 840 Shijo-cho Kashihara City, Nara, 6348522 Japan; 2 Department of Anesthesiology, Nara Medical University, 840 Shijo-cho Kashihara City, Nara, 6348522 Japan; 3 Division of Central Clinical Laboratory, Nara Medical University, 840 Shijo-cho Kashihara City, Nara, 6348522 Japan

## Abstract

Constant-voltage and constant-current stimulators may be used for transcranial electrical stimulation of motor evoked potentials (TES-MEP). However, no previous report has determined whether the two monophasic stimulation methods lead to similar responses during intra-operative monitoring. We studied differences in the lateralities of compound muscle action potentials (CMAPs) during intra-operative spinal cord monitoring via TES-MEP using monophasic constant-current and constant-voltage stimulations. CMAPs were bilaterally recorded from the upper and lower limb muscles in 95 patients who underwent elective spine and spinal cord surgery. We used two monophasic stimulation patterns: pattern 1, right anode and left cathode; pattern 2, right cathode and left anode. There were no statistically significant differences between the right and left sides with respect to success rates, wave amplitudes, and efficiencies, with constant-voltage stimulation, however, there were statistically significant differences between the right and left sides with constant-current stimulation. In case of our stimulation condition, there were no statistically significant differences between the right and left sides with respect to CMAPs with constant-voltage stimulation; constant-current stimulation was influenced by the type of monophasic stimulation, which necessitates the switch the polarity of the stimulation to bilaterally record CMAPs.

## Introduction

Iatrogenic spinal cord injury is the most serious complication in spine surgery. Transcranial electrical stimulation of motor-evoked potentials (TES-MEP), an intraoperative spinal cord monitoring technique, is an essential tool used to avoid iatrogenic spinal cord injury during risky surgeries, such as scoliosis surgery, spinal cord tumour surgery as well as severe spinal canal stenosis due to degenerative spondylotic changes. TES-MEP can be used to intraoperatively monitor motor function by recording compound muscle action potentials (CMAPs) from target muscles of the upper and lower limbs^1–4^. Previously, we have found that, in several instances, constant-voltage stimulation proves better than constant-current stimulation in terms of successfully evoking CMAPs^5^. Measuring CMAPs evoked by transcranial brain stimulation is minimally invasive and can be used to assess motor-related pathways connecting the motor cortex to the target muscle^6^. CMAP monitoring can also be used to establish the functions of the anterior horn cells and nerve roots in the spinal cord segment innervating the target muscle.

Transcranial stimulation involves an anode and a cathode placed on opposite sides of the body. It predominantly stimulates the brain on the anode side and evokes large potentials in contralateral muscles^4,7,8^. It may therefore be necessary to switch the anode-cathode laterality to record adequate muscle responses on both sides. However, to the best of our knowledge, no previous study has examined whether the anode-cathode laterality should be switched during spine surgery.

Generally, TES-MEP can be conducted with constant-voltage stimulators, which adjust the current to maintain the voltage, or constant-current stimulators, which adjust the voltage to maintain the current. Although, in several instances, we have found that constant-voltage stimulation proved better than constant-current stimulation in terms of successfully evoking CMAPs, no previous study has reported whether the methods generate responses equivalent to monophasic stimulation during intraoperative monitoring. However, this information is important for surgeons, providing knowledge of the weak point on intraoperative spinal cord monitoring.

The purpose of this study was to clarify the differences between the anode side and cathode side regarding CMAPs from target muscles of the upper and lower limbs by using monophasic TES-MEP with both constant-current and constant-voltage stimulators for intraoperative spinal cord monitoring in the same patient groups.

## Methods

This prospective within-subjects study was approved by the Nara Medical University Ethics Committee and conducted in accordance with the principles of the Declaration of Helsinki. All patients provided written informed consent.

### Subjects

The potential subject pool consisted of 258 patients who underwent elective spine and spinal cord surgeries with TES-MEP-based monitoring of motor function between July 2014 and April 2016. Of these, 141 declined to participate. Both constant-voltage and constant-current stimulators were used for 117 patients. However, for 22 patients, the available clinical data were insufficient, consisting of only one monophasic stimulation pattern (the anode and cathode on the right and left sides or vice versa). We therefore ultimately included 95 patients (50 men and 45 women; 14 to 85 years old, mean: 61 years, standard deviation: 17 years). The patients were diagnosed with cervical spinal stenosis (n = 26), cervical ossification of the posterior longitudinal ligament (n = 3), cervical tumours (n = 2), lumbar canal stenosis (n = 32), lumbar spondylolisthesis (n = 4), lumbar spinal tumours (n = 3), thoracic tumours (n = 7), scoliosis (n = 3), or other spinal disorders (n = 15). Preoperative mild-to-severe motor weakness (manual muscle test score: 0−3) of any muscle was present in 21 patients.

### Anaesthesia

To minimize the suppressive effects of anaesthetics and neuromuscular blockers on MEP waveforms, anaesthesia was standardized for all patients. No medication was administered beforehand. Fentanyl, remifentanil, and propofol were used both to induce (doses: 2–4 μg/kg, 0.25–0.5 μg/[kg·min], and 3.0–5.0 μg/mL, respectively) and maintain (doses: 2–4 μg/kg, 0.20–0.5 μg/[kg·min], and 2.0–3.0 μg/mL, respectively) anaesthesia. These drugs were delivered via a TE-371 target-controlled infusion pump (Terumo, Tokyo, Japan). The depth of anaesthesia was adjusted to maintain a bispectral index of 40–60. After induction, 0.6 mg/kg of rocuronium was administered to facilitate tracheal intubation. No additional neuromuscular blockers were administrated after tracheal intubation to avoid pharmacological reduction of the MEP waveforms. We performed a train-of-four monitoring at the time of control MEP recordings, and if the ratio of the fourth response to the first response was not at least 0.80, then sugammadex was administrated to reverse the profound residual rocuronium-induced neuromuscular blockade before CMAP recordings were initiated. After tracheal intubation, the lungs were mechanically ventilated to maintain an end-tidal CO_2_ partial pressure of 30–40 mmHg. A mixture of air and oxygen was administered. The rectal temperature was maintained at 35.5–37.0 °C. The degree of anaesthesia, end-tidal CO_2_ partial pressure, as well as the body temperature may affect spinal cord monitoring^9–12^. Therefore, these factors should be monitored intraoperatively.

### Stimulators

**Figure 1:**
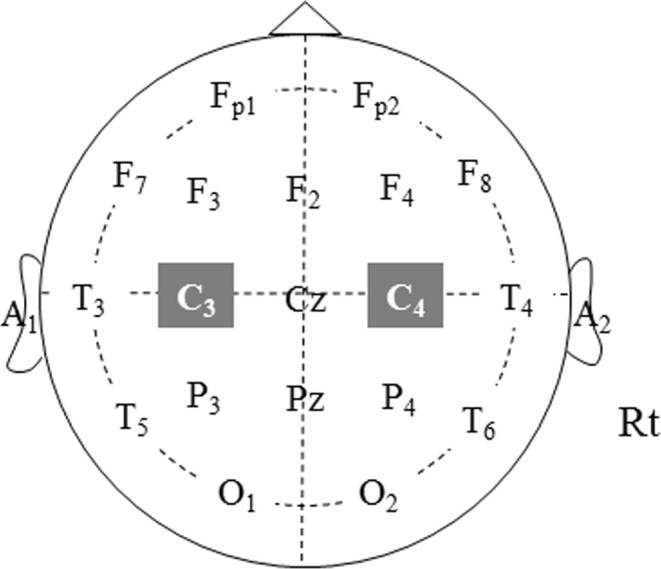
Anatomical locations according to the international 10–20 system.

The parameters used for each stimulation type are shown in Table 1. The constant-voltage stimulator used was SEN-4100 (Nihon Koden, Tokyo, Japan), and the constant-current stimulator was MS-120B (Nihon Koden). On the constant voltage stimulator, we recorded the current delivered at each stimulation time. Monophasic stimulation was delivered via anodal and cathodal stimulating electrodes that consisted of a pair of 14.5-mm silver-plated disc electrodes affixed to the C3 and C4 locations of the international 10–20 system (Fig. 1) with a conductive paste. Briefly, a five-pulse stimulation train was delivered at 500 Hz with 2-ms interstimulus intervals. At the beginning of the TES-MEP recording, the stimulus intensity was set to 500 V and 200 mA, which were the maximal settings for both devices.

**Table 1:** The conditions of the stimulator set-ups at our hospital.

	Constant Current	Constant Voltage
Stimulus count	5	5
Number of trains	1	1
Stimulus interval	2 msec	2 msec
Stimulus rate	500 Hz	500 Hz
Record time	100–200 msec	100–200 msec
Stimulus duration time	0.2 msec	50 μsec
Filter	2–3 kHz	2–3 kHz
Stimulus	200 mA	500 V

### CMAP recording

CMAPs were bilaterally recorded from the skin over the abductor pollicis brevis, deltoid, abductor hallucis, tibialis anterior, gastrocnemius, and quadricep muscles using a Neuromaster MEE1232 intraoperative TES-MEP measurement system (Nihon Koden). A ground electrode was placed on the left or right forearm, 10 cm from the olecranon.

### Study protocol

**Figure 2:**
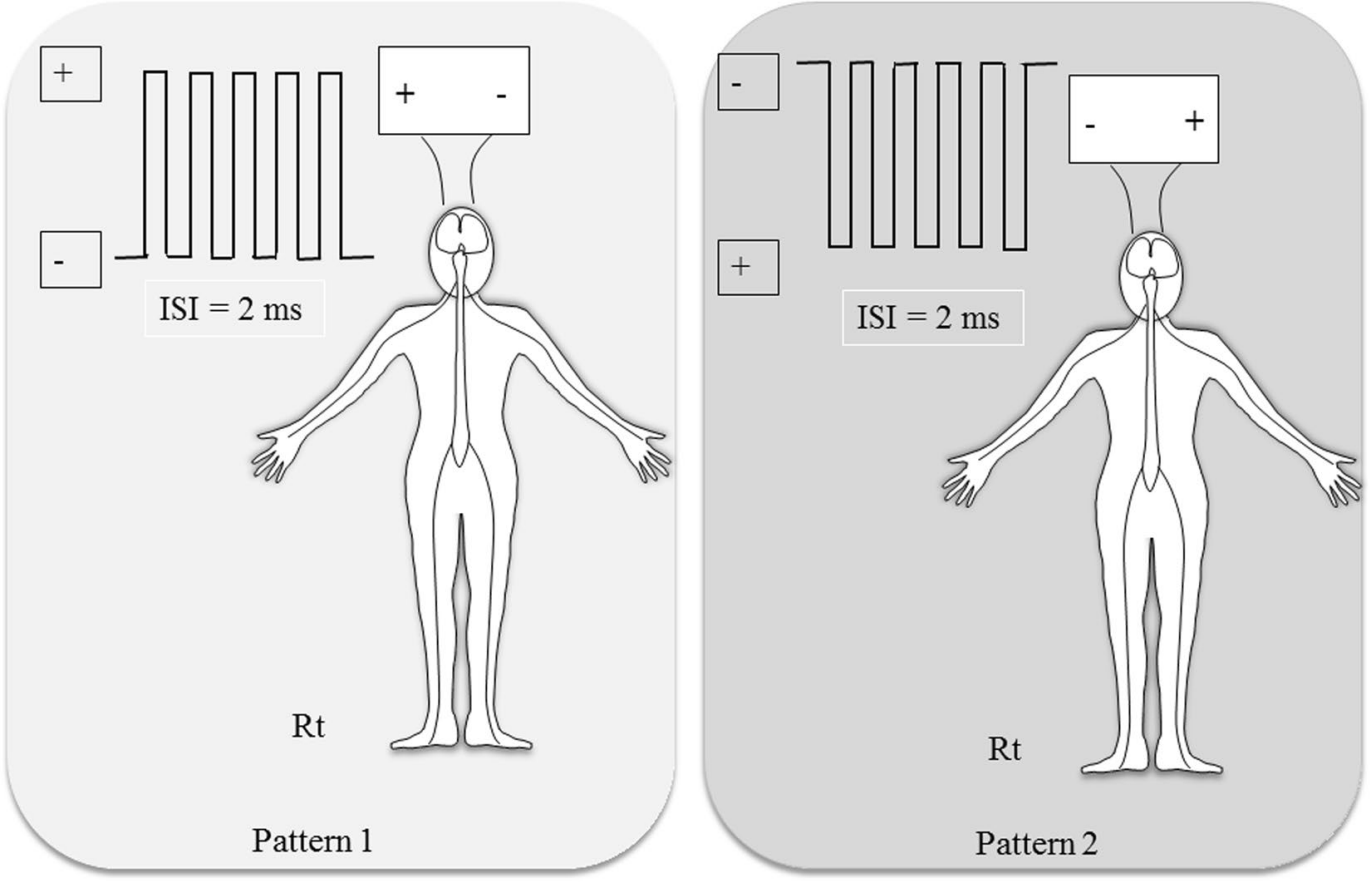
Monophasic transcranial electrical stimulation of motor-evoked potentials. Pattern 1: right anode, left cathode; Pattern 2: right cathode, left anode.

We used two monophasic stimulation patterns. In pattern 1, we placed the anode and cathode on the right and left sides, respectively, and in pattern 2, the direction of electrode placement was opposite (Fig. 2). For each patient, we used both constant-voltage and constant-current stimulators with both stimulation patterns, for four conditions in total. We recorded baseline CMAPs in the first trial after the effects of rocuronium had disappeared. We applied the four conditions in random order. The interval between the CMAP recordings was at least 2 min to avoid residual effects from the previous stimulation. TES-MEP recordings were considered successful when the recorded amplitudes of CMAPs were greater than 50 µV^13^.

We also calculated the delivered charge. The delivered charge was fixed for the constant-current cases at 200 μC (=200 (mA) × 0.2 (msec) × 5 (pulse stimulation train)). Meanwhile, we calculated the delivered charge for the constant-voltage cases: the delivered charge (μC) = ## (mA) × 0.05 (msec) × 5 (pulse stimulation train). Because it was difficult to standardize the degree of stimulation between constant-current and constant-voltage stimulations, we calculated the efficiency, the value of which was the wave amplitude for each muscle divided by the delivered charge, to standardize the obtained data from constant-current and constant-voltage stimulations.

### Statistical analysis

Statistical analyses were performed using SPSS version 17.0 (IBM, Armonk, NY). For each analysed muscle, the success rates for baseline recordings obtained with constant-voltage or constant-current stimulators were compared with chi-squared tests, while the wave amplitudes were compared using Wilcoxon signed-rank tests. For the delivered-charge comparisons, we used the Student’s t-test. Statistical significance was defined as p < 0.05.

Our study was registered in the Japan Primary Registries Network (registration number: UMIN000032651). The registration date was 20/05/2018.

### Ethical approval

All procedures performed in studies involving human participants were in accordance with the ethical standards of the institutional research committee and with the 1964 Helsinki declaration and its later amendments or comparable ethical standards.

## Results

### Calculation of total charge delivered

As mentioned above, the delivered charge was fixed for the constant-current cases at 200 μC.

The delivered current for the constant-voltage cases was 1028.5 ± 147.8 mA on pattern 1 and 1038.6 ± 149.2 mA on pattern 2. The calculated delivered charge was 257.1 ± 36.9 μC (95% CI: 249.6–264.6) on pattern 1 and 259.7 ± 37.3 μC (95% CI: 252.1–267.3) on pattern 2 for the constant-voltage cases.

There were statistically significant differences with respect to the delivered charge between constant-voltage and constant-current stimulations with pattern 1 and pattern 2 (p < 0.01).

### Success rates for each stimulation pattern in individual muscles

When using pattern 1, we found that the constant-current stimulator produced significantly higher success rates in the left-side muscles than in the right-side muscles, but with the constant-voltage stimulator, we did not observe statistically significant differences between the right side and left side with respect to the success rates (Table 2). When comparing the success rates obtained with the constant-current stimulator to those obtained with the constant-voltage stimulator in pattern 1, we found no significant differences in the left-side muscles, except for the left abductor hallucis (p = 0.03), where we observed a significantly higher success rate with the constant-voltage stimulator. However, we found significantly higher success rates with the constant-voltage stimulator in all the right-side muscles (Table 2).

**Table 2:** Success rates of detectable CMAPs (constant current versus constant voltage) during stimulation.

Stimulation		Wave amplitude	Upper limbs				Lower limbs							
Pattern			L-APB	R-APB	L-Del	R-Del	L-AH	R-AH	L-TA	R-TA	L-GC	R-GC	L-Quad	R-Quad
Pattern 1	Constant-current	Less than/equal to 50 μV (cases)	5	28	16	27	7	26	20	45	24	53	42	62
		More than 50 μV (cases)	90	67	79	68	88	69	75	50	71	42	53	33
	Success rate (%)		94.7	70.5	83.2	71.6	92.6	72.6	78.9	52.6	74.7	44.2	55.8	34.7
	Right v. left success rate p values		<0.01		0.057		<0.01		<0.01		<0.01		<0.01	
right: anode, left cathode	Constant-voltage	Less than/equal to 50 μV (cases)	7	4	10	8	1	4	11	10	15	19	29	34
		More than 50 μV (cases)	88	91	85	87	94	91	84	85	80	76	66	61
	Success rate (%)		92.6	95.8	89.5	91.6	98.9	95.8	88.4	89.5	84.2	80.0	69.5	64.2
	Right v. left success rate p values		0.35		0.62		0.17		0.82		0.45		0.44	
	Constant-current v. constant-voltage success rate p values		0.6	<0.01	0.2	<0.01	0.03	<0.01	0.1	<0.01	0.1	<0.01	0.1	<0.01
Pattern 2	Constant-current	Less than/equal to 50 μV (cases)	26	10	34	14	32	15	47	26	52	26	64	51
		More than 50 μV (cases)	69	85	61	81	63	80	48	69	43	69	31	44
	Success rate (%)		72.6	89.5	64.2	85.3	66.3	84.2	50.5	72.6	45.3	72.6	32.6	46.3
	Right v. left success rate p values		<0.01		<0.01		<0.01		<0.01		<0.01		0.054	
right: cathode, left: anode	Constant-voltage	Less than/equal to 50 μV (cases)	7	4	6	5	3	2	14	16	17	16	28	36
		More than 50 μV (cases)	88	91	89	90	92	93	81	79	78	79	67	59
	Success rate (%)		92.6	95.8	93.7	94.7	96.8	97.9	85.3	83.2	82.1	83.2	70.5	62.1
	Right v. left success rate p values		0.35		0.76		0.65		0.69		0.85		0.22	
	Constant-current v. constant-voltage p values		<0.01	0.10	<0.01	0.03	<0.01	0.001	<0.01	0.08	<0.01	0.08	<0.01	0.03

CMAP: compound muscle action potential, Del: deltoid, APB: abductor pollicis brevis, Quad: quadriceps, TA: tibialis anterior, GC: gastrocnemius, AH: abductor halluces, L: left, R: right.

When using pattern 2, we found that the constant-current stimulator produced significantly higher success rates in the right-side muscles than in the left-side muscles, but with the constant-voltage stimulator, we did not observe statistically significant differences between right side and left side with respect to the success rates (Table 2). When comparing the success rates obtained with the constant-current stimulator to those obtained with the constant-voltage stimulator in pattern 2, we found no significant differences in the right-side muscles, except for the right deltoid (p = 0.03), abductor hallucis (p = 0.001), and quadriceps (p = 0.03), where we observed a significantly higher success rate with the constant-voltage stimulator. However, we found significantly higher success rates with the constant-voltage stimulator in all the right-side muscles.

### Wave amplitudes in individual muscles during stimulation with patterns 1 and 2

When using pattern 1, we found that the constant-current stimulator produced consistently higher CMAP amplitudes in left-side muscles than in right-side muscles, but when using pattern 2, we found that the constant-current stimulator produced consistently higher CMAP amplitudes in right-side muscles than in left-side muscles (Table 3). With both patterns, with the constant-voltage stimulator, we did not observe statistically significant differences between right side and left side with respect to CMAP amplitudes, except in the deltoid (Table 3).

**Table 3:** Comparisons of CMAPs (constant current versus constant voltage) between the right and left sides during stimulation using patterns 1 and 2.

Muscles	Laterality	Constant current						Constant voltage					
		Pattern 1			Pattern 2			Pattern 1			Pattern 2		
		right: anode, left: cathode			right: cathode, left: anode			right: anode, left: cathode			right: cathode, left: anode		
		Median	Interquartile range	p value	Median	Interquartile range	p value	Median	Interquartile range	p value	Median	Interquartile range	p value
Del	Right	194.0	[35–602]	<0.01	430.0	[105–1155.5]	<0.01	520.0	[200–1440]	<0.01	456.0	[220–1105]	0.04
	Left	330.0	[110–819]		140.0	[18.3–349.3]		370.0	[147–830]		376.5	[141.5–1012.5]	
APB	Right	470.0	[31–1680]	<0.01	1335.0	[245.3–3197.5]	<0.01	1280.0	[240–3030]	0.46	1325.0	[254.3–2785.0]	0.09
	Left	1176.0	[304–2690]		380.0	[37.8–1572.5]		1190.0	[240–2890]		940.0	[240.5–2520.0]	
Quad	Right	10.4	[0–133]	<0.01	47.0	[5–281]	<0.01	101.0	[16.1–560]	0.51	80.0	[16.6–460]	0.60
	Left	75.0	[0–320]		10.3	[0–93]		150.0	[27–423.0]		139.0	[20–530]	
TA	Right	67.0	[0–390]	<0.01	280.0	[34–970]	<0.01	580.0	[136–2300]	0.02	522.0	[130–1910]	0.58
	Left	338.0	[64–1490]		54.0	[0–430]		630.0	[100–1780]		490.0	[148–1790]	
GC	Right	42.0	[0–193]	<0.01	140.0	[41–570]	<0.01	250.0	[85–865]	0.63	266.0	[83–720]	0.33
	Left	220.0	[50–620]		32.0	[0–212]		340.0	[83–880]		349.0	[88–967]	
AH	Right	160.0	[50–630]	<0.01	555.0	[112–1600]	<0.01	1020.0	[360–2032]	0.37	1030.0	[423–2210]	0.43
	Left	830.0	[210–1700]		180.0	[20–750]		1150.0	[443–2400]		1160.0	[410–2061]	

CMAP: compound muscle action potential, Del: deltoid, APB: abductor pollicis brevis, Quad: quadriceps, TA: tibialis anterior, GC: gastrocnemius, AH: abductor halluces.

### Efficiency for each muscle during stimulation with patterns 1 and 2

When using pattern 1, we found that the constant-current stimulator showed consistently higher efficiency with respect to the left-side muscles than right-side muscles, but when using pattern 2, we found that the constant-current stimulator showed consistently higher efficiency with respect to the right-side muscles than in left-side muscles (p < 0.01). With both patterns, with the constant-voltage stimulator, we did not observe statistically significant differences between right side and left side in terms of efficiency (p > 0.05) (Table 4).

**Table 4:** Efficiencies of stimulation (constant current versus constant voltage).

muscles	laterality	constant current						constant voltage					
		Pattern 1			Pattern 2			Pattern 1			Pattern 2		
		right: anode, left: cathode			right: cathode, left: anode			right: anode, left: cathode			right: cathode, left: anode		
		Median	interquartile range	p value	Median	interquartile range	p value	Median	interquartile range	p value	Median	interquartile range	p value
Del	Right	0.97	[0.18–3.01]	0.05	2.15	[0.53–5.72]	<0.01	2.11	[0.83–5.04]	0.06	1.94	[0.85–3.98]	0.19
	Left	1.65	[0.55–4.10]		0.70	[0.07–1.70]		1.44	[0.53–3.31]		1.48	[0.55–3.81]	
APB	Right	2.35	[0.16–8.40]	<0.01	6.35	[1.24–1.57]	<0.01	4.43	[0.84–11.31]	0.89	5.19	[1.00–10.58]	0.61
	Left	5.88	[1.52–13.45]		1.55	[0.19–7.65]		4.82	[0.90–11.69]		3.73	[0.96–9.93]	
Quad	Right	0.05	[0–0.67]	<0.01	0.24	[0.03–1.41]	<0.01	0.38	[0.06–2.38]	0.88	0.35	[0.07–1.97]	0.69
	Left	0.38	[0–1.60]		0.05	[0–0.47]		0.59	[0.11–1.50]		0.56	[0.08–1.99]	
TA	Right	0.34	[0–1.95]	<0.01	0.24	[0.03–1.41]	<0.01	2.34	[0.52–8.62]	0.60	2.08	[0.51–7.32]	1.00
	Left	1.69	[0.32–7.45]		0.05	[0–0.47]		2.26	[0.40–6.79]		2.03	[0.57–6.01]	
GC	Right	0.21	[0–0.97]	<0.01	0.70	[0.21–2.85]	<0.01	1.02	[0.28–3.74]	0.73	1.04	[0.31–3.06]	0.56
	Left	1.10	[0.25–3.10]		0.16	[0–1.06]		1.39	[0.28–3.55]		1.50	[0.36–4.22]	
AH	Right	0.80	[0.25–3.15]	<0.01	2.78	[0.56–8.00]	<0.01	4.27	[1.24–7.86]	0.55	3.95	[1.67–8.49]	0.92
	Left	4.15	[1.05-8.50]		0.90	[0.10–3.75]		4.50	[1.59–9.52]		4.27	[1.62–8.40]	

Del: deltoid, APB: abductor pollicis brevis, Quad: quadriceps, TA: tibialis anterior, GC: gastrocnemius, AH: abductor halluces, L: left, R: right.

The case illustrated below is representative of the results obtained in the subjects.

### Illustrative case

**Figure 3:**
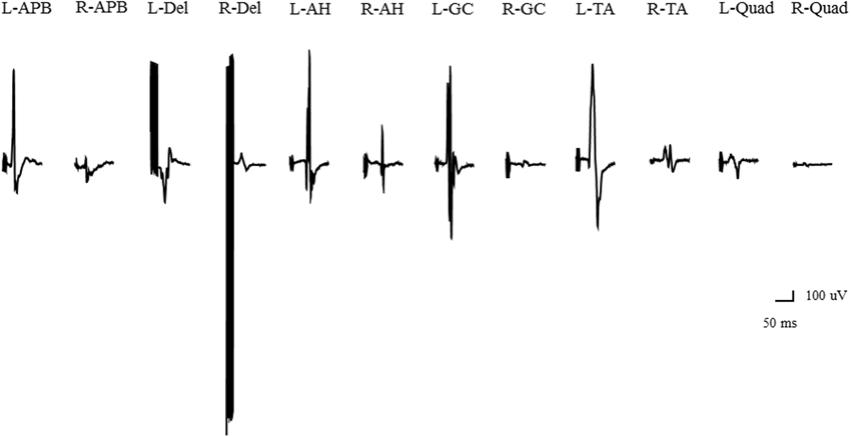
Wave amplitudes obtained using different stimulation patterns. Wave amplitudes with constant-current stimulation and pattern 1. Abbreviations: AH, abductor hallucis; APB, abductor pollicis brevis; Del, deltoid; GC, gastrocnemius; L, left; Quad, quadriceps; R, right; TA, tibialis anterior.

**Figure 4:**
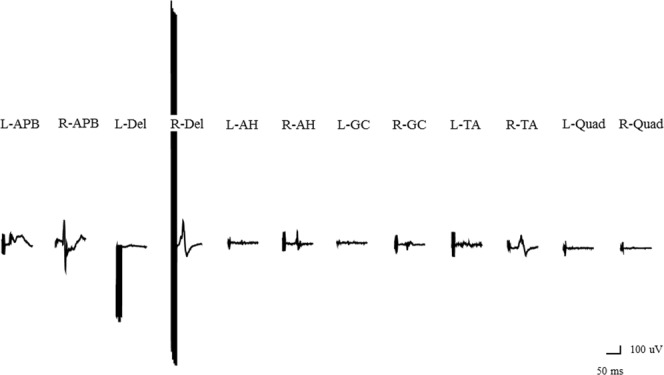
Wave amplitudes with constant-current stimulation and pattern 2. Abbreviations: AH, abductor hallucis; APB, abductor pollicis brevis; Del, deltoid; GC, gastrocnemius; L, left; Quad, quadriceps; R, right; TA, tibialis anterior.

**Figure 5:**
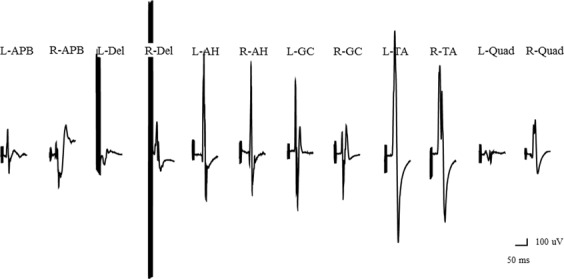
Wave amplitudes with constant-voltage stimulation and pattern 1. Abbreviations: AH, abductor hallucis; APB, abductor pollicis brevis; Del, deltoid; GC, gastrocnemius; L, left; Quad, quadriceps; R, right; TA, tibialis anterior.

**Figure 6:**
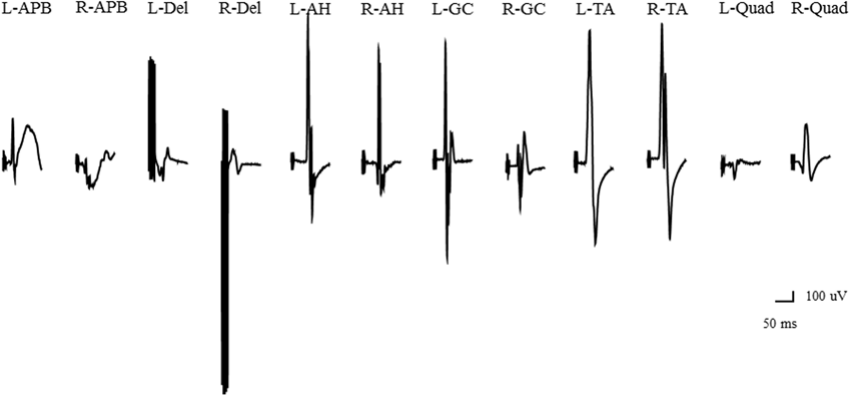
Wave amplitudes with constant-voltage stimulation and pattern 2. Abbreviations: AH, abductor hallucis; APB, abductor pollicis brevis; Del, deltoid; GC, gastrocnemius; L, left; Quad, quadriceps; R, right; TA, tibialis anterior.

An 81-year-old man had scoliosis and lumbar kyphosis. He did not exhibit any neurological deficits before surgery. Constant-current stimulation produced greater wave amplitudes on the side contralateral to the anode than on the side ipsilateral to it (Figs 3 and 4), but with constant-voltage stimulation, we did not observe significant differences between right side and left side in terms of wave amplitudes (Figs 5 and 6).

## Discussion

Many previous reports have highlighted the importance of MEP monitoring during spinal surgeries^14,15^, and TES-MEP is regarded as the gold standard^7,14,16^. TES-MEP is relatively non-invasive and can be used to record CMAPs from numerous muscles in the upper and lower limbs. It also enables separate recordings from muscles on each side, which allows us to analyse laterality. This is convenient in the case of monophasic stimulation, as it may be necessary to switch the anode-cathode laterality in order to bilaterally analyse CMAPs intraoperatively. In contrast, biphasic stimulation, which is another reversed-phase stimulation method, can stimulate both sides of the brain almost simultaneously. This method enables the bilateral evaluation of spinal cord functions with a train of biphasic pulse stimuli without the need to reverse the polarity. Ukegawa *et al*.^17^ have shown that biphasic stimulation with a constant-current stimulator is an effective TES-MEP monitoring method and may reduce measurement time. However, they only compared monophasic and biphasic stimulation with a constant-current stimulator and did not report on the effects of constant-voltage stimulators or monophasic stimulation. We previously reported that constant-voltage stimulation was superior in terms of achieving adequate wave amplitude to maintain intraoperative spinal cord monitoring than constant-current stimulation. However, we did not determine the differences of laterality under monophasic stimulation between constant-voltage and constant-current stimulations at that time.

Here, we compared the success rates with respect to CMAP wave amplitudes and efficiencies obtained with monophasic stimulation using a constant-current stimulator to those obtained with a constant-voltage stimulator. With the constant-voltage stimulator, we did not observe statistically significant differences between right side and left side in terms of success rates, wave amplitudes, as well as efficiencies but with the constant-current stimulator, significantly greater success rates, wave amplitudes, and efficiencies were observed on the side contralateral to the anode. We therefore conclude that when using constant-current stimulators, it is necessary to switch the stimulation polarity to study muscle responses bilaterally in order to avoid the disadvantages of monophasic stimulation. Alternatively, it may be necessary to use biphasic stimulation^17^. We believe that this information is important for surgeons to maintain adequate intraoperative spinal cord monitoring to prevent spinal cord injury during surgery.

To our knowledge, this study is the first to compare the success rate of laterality with constant-current and constant-voltage stimulators in monophasic TES-MEP stimulation. Constant-voltage stimulation enabled us to bilaterally record CMAPs. This may be because constant-voltage stimulators act on deep pyramidal tracts beyond the pyramidal decussation, but we have no data to support the above statement. This is one limitation of our study.

Our study has several other limitations. First, our cases comprised patients with several different kinds of spinal disorders, with pathologies distributed over the entire spinal cord. Although this may broaden the applicability of our results, it introduces several potentially confounding factors that may have hindered the identification of differences between the techniques. Second, we studied only a few cases, mostly due to patient preferences in our potential subject pool. Third, we only analysed three variables: success rates, wave amplitudes, and efficiencies. Although it is potentially important to evaluate the degree to which the stimulation reaches the pyramidal tract, it was not clinically feasible in this study. Fourth, we used maximal stimulation levels for both stimulators. Although we found that constant-voltage stimulation outperformed constant-current stimulation in terms of successfully bilaterally evoking CMAPs, it is conceivable that the constant-voltage stimulation was simply more supramaximal than the constant-current stimulation. However, we could not elevate constant-current stimulation beyond 200 mA owing to the limitations of the device. Fifth, we placed the stimulating electrodes on C3 and C4 and did not test any other placements despite Legatt *et al*.^8^ having reported that the Cz/Fz stimulating montage may more reliably bilaterally stimulate the lower limb motor pathways following a single stimulation. Additional studies are necessary to address these limitations. Sixth, anatomical and physiological aspects may affect the results of electrode laterality, although we recorded CMAPs from the upper and lower limb muscles under the same conditions in each subject. We believe that the anatomical and physiological aspects had a very small effect.

## Conclusions

TES-MEP monitoring during spinal cord surgery can be achieved with either a constant-voltage stimulator or a constant-current stimulator. Here, we did not find statistically significant differences with respect to success rates, CMAP wave amplitudes, as well as efficiencies between the anode-cathode laterality with the constant-voltage stimulator (500V). In contrast, the results obtained with the constant-current stimulator (200 mA) were influenced by the anode-cathode laterality, such that greater success rates, CMAP wave amplitudes, and efficiencies were observed on the side contralateral to the anode. It is therefore necessary to switch the polarity of the stimulation to bilaterally record CMAPs.

## Supplementary information

research protocol layout

acknowledgements
