# Supplementary material for: Monophasic transcranial constant-current versus constant-voltage stimulation of motor-evoked potentials during spinal surgery
Source: Sci Rep. 2019 Mar 7;9:3773. doi: 10.1038/s41598-019-39883-y (PMC6405953; doi:10.1038/s41598-019-39883-y)
Supplement: Supplementary file 1 — research protocol layout [file 41598_2019_39883_MOESM1_ESM.docx]

Research Protocol

Title: Monophasic transcranial constant-current versus constant-voltage stimulation of motor-evoked potentials during spinal surgery

No sponsor/no funder

Hideki Shigematsu

Nara Medical University, 840 Shijo-cho Kashihara Nara Japan

**Study protocol**

Constant-voltage and constant-current stimulators may be used for transcranial electrical stimulation of motor evoked potentials (TES-MEP). However, no previous reports have determined whether the two monophasic stimulation methods lead to similar responses during intra-operative monitoring. We studied differences in the literalities of compound muscle action potentials (CMAPs) during intra-operative spinal cord monitoring via TES-MEP using monophasic constant-current and constant-voltage stimulations.

We used two monophasic stimulation patterns. In pattern 1, we placed the anode and cathode on the right and left sides, respectively, and in pattern 2, we did the opposite. For each patient, we used both constant-voltage and constant-current stimulators with both stimulation patterns, for four conditions in total. We recorded baseline CMAPs in the first trial after the effects of rocuronium had disappeared. We applied the four conditions in random order. The interval between CMAP recordings was at least 2 min to avoid residual effects from the previous stimulation. TES-MEP recordings were considered successful when the recorded amplitudes of CMAPs were greater than 50 µV .

We also calculated the delivered charge. The delivered charge was fixed for the constant-current cases at 200µC (=200 (mA) × 0.2 (msec) × 5 (pulse stimulation train)). Meanwhile, we calculated the delivered charge for the constant-voltage cases: the delivered charge (µC)= ## (mA) × 0.05 (msec) ×5 (pulse stimulation train). Because it was difficult to standardize the degree of stimulation between constant-current and constant-voltage stimulations, we calculated the efficiency, the value of which was wave amplitude for each muscle divided by the delivered charge, to standardize the obtained data from constant-current and constant-voltage stimulations.

*Expected outcomes:* constant current and constant voltage stimulation lead to similar responses during intra-operative monitoring.
