# Supplementary material for: Monophasic transcranial constant-current versus constant-voltage stimulation of motor-evoked potentials during spinal surgery
Source: Sci Rep. 2019 Mar 7;9:3773. doi: 10.1038/s41598-019-39883-y (PMC6405953; doi:10.1038/s41598-019-39883-y)
Supplement: Supplementary file 2 — acknowledgements [file 41598_2019_39883_MOESM2_ESM.docx]

**Acknowledgements:** The authors thank Sayomi Yamamoto, Junko Kato, Kazuya Moriwake, Kiyomi Mameda, Ryohei Mizobata, and Ryo Nakamori for their technical assistance with TES-MEP.
